# Supplementary material for: Niche-specific dermal macrophage loss promotes skin capillary ageing
Source: Nature. 2025 Oct 15;648(8092):173–81. doi: 10.1038/s41586-025-09639-y (PMC12675291; doi:10.1038/s41586-025-09639-y)
Supplement: Supplementary file 1 — A guide to Supplementary Videos 1–6. [file 41586_2025_9639_MOESM1_ESM.pdf]

nature portfolio

https://doi.org/10.1038/s41586-025-09639-y

Supplementary information

Niche-specific dermal macrophage loss promotes skin capillary ageing

In the format provided by the authors and unedited

Supplemental Information Guide

Authors and Affiliations

1. Department of Cell Biology, New York University School of Medicine, New York, NY 10016, USA
   Kailin R. Mesa, Kevin A. O'Connor, Alexandra Dolynuk, Michelle Rivera Lomeli, & Dan R. Littman

2. Department of Pathology and Laboratory Medicine, Weill Cornell Medicine, New York-Presbyterian Hospital, New York, NY 10021, USA
   Charles Ng & Steven P. Salvatore

3. Perlmutter Cancer Center, New York University Langone Health, New York, NY 10016, USA
   Dan R. Littman

4. Howard Hughes Medical Institute, New York, NY 10016, USA
   Dan R. Littman

Supplemental Video 1-6

Supplemental Video 1. Serial optical sections through mouse plantar skin, including epidermis (0-25μm), upper dermis (26-50μm), and lower dermis (51-100μm) in *R26-mTmG* (red) mice. Note Second and Third Harmonic Generation illuminates dermal collagen (blue) and red blood cells (white), respectively. Scale bar, 50μm.

Supplemental Video 2. Serial optical sections through mouse plantar skin, including epidermis (0-15μm), upper dermis (16-40μm), and lower dermis (41-52μm) in *Csf1r-EGFP* mice. Note distinct macrophage (green) density and morphology in each tissue niche. Scale bar, 50μm.

Supplemental Video 3. Time-lapse recording of capillary blood flow via third harmonic generation (white) from red blood cells and intravenous rhodamine dextran (red) in *Csf1r-EGFP* mice. Note some capillary segments show obstructed red blood cell and rhodamine dextran flow. Scale bar, 50μm.

Supplemental Video 4. Serial optical sections through upper dermal capillary niche in *Cx3cr1-GFP; R26-mTmG* mice. Note macrophage (green) localization and morphology around capillary (red) network. Scale bar, 50μm.

Supplemental Video 5. Time-lapse recording of capillary blood flow in *Cx3cr1-CreER; R26-mTmG* mice. Note inconsistent and obstructed blood flow (white) in capillary (red) segments without associated macrophages (green). Scale bar, 50μm.

Supplemental Video 6. Time-lapse recording following laser-induced capillary clot formation in *Csf1r-GFP; R26-mTmG* mice. Note rapid migration of neighboring capillary-associated macrophages (green) toward site of clot formation (yellow arrowhead). Scale bar, 50μm.
