# Supplementary material for: Niche-specific dermal macrophage loss promotes skin capillary ageing
Source: Nature. 2025 Oct 15;648(8092):173–81. doi: 10.1038/s41586-025-09639-y (PMC12675291; doi:10.1038/s41586-025-09639-y)
Supplement: Supplementary file 2 — Reporting Summary [file 41586_2025_9639_MOESM2_ESM.pdf]

Corresponding author(s): Kailin R Mesa
Dan R Littman

Last updated by author(s): Jul 6, 2025

Reporting Summary

Nature Portfolio wishes to improve the reproducibility of the work that we publish. This form provides structure for consistency and transparency in reporting. For further information on Nature Portfolio policies, see our Editorial Policies and the Editorial Policy Checklist.

Statistics

For all statistical analyses, confirm that the following items are present in the figure legend, table legend, main text, or Methods section.

n/a	Confirmed	
☐	☒	The exact sample size (*n*) for each experimental group/condition, given as a discrete number and unit of measurement
☐	☒	A statement on whether measurements were taken from distinct samples or whether the same sample was measured repeatedly
☐	☒	The statistical test(s) used AND whether they are one- or two-sided<br>*Only common tests should be described solely by name; describe more complex techniques in the Methods section.*
☐	☒	A description of all covariates tested
☐	☒	A description of any assumptions or corrections, such as tests of normality and adjustment for multiple comparisons
☐	☒	A full description of the statistical parameters including central tendency (e.g. means) or other basic estimates (e.g. regression coefficient) AND variation (e.g. standard deviation) or associated estimates of uncertainty (e.g. confidence intervals)
☐	☒	For null hypothesis testing, the test statistic (e.g. *F*, *t*, *r*) with confidence intervals, effect sizes, degrees of freedom and *P* value noted<br>*Give P values as exact values whenever suitable.*
☒	☐	For Bayesian analysis, information on the choice of priors and Markov chain Monte Carlo settings
☒	☐	For hierarchical and complex designs, identification of the appropriate level for tests and full reporting of outcomes
☒	☐	Estimates of effect sizes (e.g. Cohen's *d*, Pearson's *r*), indicating how they were calculated

Our web collection on statistics for biologists contains articles on many of the points above.

Software and code

Policy information about availability of computer code

Data collection	Fluoview FV32S-SW (Olympus v2.3.l.163), Aperio ImageScope (Leica, v12.4.6), FACSDiva (BD Biosciences)
Data analysis	Fiji (NIH v2.14.0), Prism (GraphPad, v10.0.2), FlowJo (BD Biosciences, 10.10.1)

For manuscripts utilizing custom algorithms or software that are central to the research but not yet described in published literature, software must be made available to editors and reviewers. We strongly encourage code deposition in a community repository (e.g. GitHub). See the Nature Portfolio guidelines for submitting code & software for further information.

Data

Policy information about availability of data

All manuscripts must include a data availability statement. This statement should provide the following information, where applicable:

- Accession codes, unique identifiers, or web links for publicly available datasets
- A description of any restrictions on data availability
- For clinical datasets or third party data, please ensure that the statement adheres to our policy

Source data will be provided with this paper. All other data supporting the findings of this study are available from the corresponding authors on reasonable request.

Research involving human participants, their data, or biological material

Policy information about studies with human participants or human data. See also policy information about sex, gender (identity/presentation), and sexual orientation and race, ethnicity and racism.

Reporting on sex and gender	Clinical information and laboratory data were obtained from the electronic medical record. Sex and gender information was not used.
Reporting on race, ethnicity, or other socially relevant groupings	N/A
Population characteristics	Patients in young group were below 40 years of age (19 - 37 years old). Patients in old group were above 40 years of age (79 - 97 years old). Patients with skin or vascular pathologies were excluded.
Recruitment	Written informed consent was obtained for postmortem examination from next of kin for all patients.
Ethics oversight	Study protocol was approved by Weill Cornell Department of Pathology

Note that full information on the approval of the study protocol must also be provided in the manuscript.

Field-specific reporting

Please select the one below that is the best fit for your research. If you are not sure, read the appropriate sections before making your selection.

☒ Life sciences ☐ Behavioural & social sciences ☐ Ecological, evolutionary & environmental sciences

For a reference copy of the document with all sections, see nature.com/documents/nr-reporting-summary-flat.pdf

Life sciences study design

All studies must disclose on these points even when the disclosure is negative.

Sample size	Three or more mice per group were used in each experiment. The precise number of animals used are given in the figure legend. The sample size was determined from our previous experience and from what is accepted in the field.
Data exclusions	No samples were excluded from the analysis.
Replication	Experiments were replicated at least three times unless otherwise specified. All attempts were successful.
Randomization	Allocation into sample groups was random. In addition, experimental and control mice were used from the same litter whenever possible. Both males and females were used.
Blinding	For human skin samples, the age group of samples were blinded to the researcher. For mouse imaging experiments, random ROI were selected for image analysis. Quantification of CAM coverage and capillary blood flow was performed blinded to minimize researcher bias.

Reporting for specific materials, systems and methods

We require information from authors about some types of materials, experimental systems and methods used in many studies. Here, indicate whether each material, system or method listed is relevant to your study. If you are not sure if a list item applies to your research, read the appropriate section before selecting a response.

Materials & experimental systems

n/a	Involved in the study	
☐	☒	Antibodies
☒	☐	Eukaryotic cell lines
☒	☐	Palaeontology and archaeology
☐	☒	Animals and other organisms
☒	☐	Clinical data
☒	☐	Dual use research of concern
☒	☐	Plants

Methods

n/a	Involved in the study	
☒	☐	ChIP-seq
☐	☒	Flow cytometry
☒	☐	MRI-based neuroimaging

Antibodies

Antibodies used	CD68 HRP (Clone 514H12, Leica PA0273), ERG HRP (Clone EPR3864, Abcam ab92513), CD206 AF647 (Clone C068C2, BioLegend 141712), Gr1 AF647 (Clone RB6-8C5, BioLegend 108418).
Validation	Antibodies were used according to recommendations of the manufacturer.

Animals and other research organisms

Policy information about studies involving animals; ARRIVE guidelines recommended for reporting animal research, and Sex and Gender in Research

Laboratory animals	Mice were bred and maintained in the Alexandria Center for the Life Sciences animal facility of the New York University School of Medicine, in specific pathogen-free conditions. Albino B6 (B6(Cg)-Tyrc-2J/J, Jax 000058), Csf1rEGFP (B6.Cg-Tg(Csf1r-EGFP)1Hume/J, Jax 018549), Ccr2RFP (B6.129(Cg)-Ccr2tm2.1Ifc/J, Jax 017586), R26mTmG (B6.129(Cg)-Gt(ROSA)26Sortm4(ACTB-tdTomato,-EGFP)Luo/J, Jax 007676), R26nTnG (B6N.129S6-Gt(ROSA)26Sortm1(CAG-tdTomato*,-EGFP*)Ees/J, Jax 023537), LysMCre (B6.129P2-Lyz2tm1(cre)Ifo/J, Jax 004781), Rac1f/f (Rac1tm1Djk/J, Jax 005550), and CAG-dsRed (B6.Cg-Tg(CAG-DsRed*MST)1Nagy/J, JAX 006051) mice were purchased from Jackson Laboratories. R26dsRed mice were described previously (Luche et al., 2007) and were obtained from the laboratory of Dr. Gordon Fishell. Cx3cr1CreER, Cx3cr1GFP, and Cx3cr1DTR were generated in our laboratory and have been described (Parkhurst, et al., 2013; Jung, et al., 2000; Diehl, et al., 2013). All experimental mice for this study were albino (homozygous for Tyrc-2J) as is required for intravital imaging in the skin. Mice 1-24 months old were used for experiments.
Wild animals	This study did not involve wild animals.
Reporting on sex	Mice from experimental and control groups were randomly selected from either sex for live imaging experiments.
Field-collected samples	This study did not involve samples collected from the field.
Ethics oversight	All animal procedures were performed in accordance with protocols approved by the Institutional Animal Care and Usage Committee of New York University School of Medicine.

Note that full information on the approval of the study protocol must also be provided in the manuscript.

Flow Cytometry

Plots

Confirm that:

☒ The axis labels state the marker and fluorochrome used (e.g. CD4-FITC).

☒ The axis scales are clearly visible. Include numbers along axes only for bottom left plot of group (a 'group' is an analysis of identical markers).

☒ All plots are contour plots with outliers or pseudocolor plots.

☒ A numerical value for number of cells or percentage (with statistics) is provided.

Methodology

Sample preparation	Peripheral blood samples were collected from the submandibular (facial) vein in tubes containing EDTA (BD Biosciences; Dipotassium EDTA Microtainer; Cat# 365972). Red blood cells were lysed with ACK lysing buffer and the remaining cells were subjected flow cytometric analysis.
Instrument	LSR II Flow Cytometer (BD Biosciences)
Software	Samples were ran on the LSR III with FACSDiva and FlowJo 10.10.1 software (BD Biosciences)
Cell population abundance	Cell population abundance was displayed as percentage of total cells from blood sample.
Gating strategy	Gates were defined from blood samples of WT and single fluorescent reporter (CAG-dsRed and CSF1R-EGFP) mouse strains.

☒ Tick this box to confirm that a figure exemplifying the gating strategy is provided in the Supplementary Information.
